# Developmental origins of pregnancy loss in cattle

**DOI:** 10.1590/1984-3143-AR2026-0066

**Published:** 2026-08-28

**Authors:** Inés Flores-Borobia, Alba Pérez-Gómez, Nuria Martínez de los Reyes, Leopoldo González-Brusi, Beatriz Galiano-Cogolludo, Carlos Irala, Alexia Siegmund-Sabater, Priscila Ramos-Ibeas, Pablo Bermejo-Álvarez

**Affiliations:** 1 Instituto Nacional de Investigación y Tecnología Agraria y Alimentaria – INIA, Consejo Superior de Investigaciones Científicas – CSIC, Animal Reproduction Department, Madrid, Spain; 2 Institut National de Recherche Pour L'agriculture, L'alimentation et L'environnement – INRAE, Centre National de la Recherche Scientifique – CNRS, Nouzilly, France; 3 University of Tours, Tours, France; 4 Agronomy Faculty, Universidad de Buenos Aires, Buenos Aires, Argentina

**Keywords:** embryonic disc, epiblast, survival, pregnancy, cattle

## Abstract

Pregnancy losses constitute a major economic burden in cattle farming and are caused by developmental failures occurring mostly during the first month of gestation. This review integrates knowledge gathered from direct and indirect estimations of embryo survival at critical developmental periods with knowledge obtained from functional *in vivo* and *in vitro* studies to elucidate the developmental origins of pregnancy loss in cattle. Fertilization failures and embryonic arrest prior to the blastocyst stage, estimated based on morphological observation of flushed structures, average 10 to 20 and 10 to 30%, respectively. Reduced oocyte competence arises as the major driver of the underlying developmental failures, including impaired embryonic genome activation, loss of totipotency and defective first lineage differentiation. To estimate embryonic losses from blastocyst to implantation, direct observation of embryo development and indirect proxies of pregnancy have been employed. Indirect proxies such as nonreturn to oestrus or biochemical markers such as Interferon Stimulated Genes or Pregnancy Associated Glycoproteins inform on the development of extra-embryonic membranes, but do not distinguish between viable pregnancies and anembryonic pregnancies, where embryo-devoid structures formed by extra-embryonic membranes are able to maintain the corpus luteus to day 37. Merging data from recovery rates of early elongated structures and incidence of embryo-devoid structures, embryonic mortality from blastocyst hatching to pregnancy recognition oscillates between 12-79% for *in vivo* derived and 40-86% for *in vitro* produced embryos. The exclusion of anembryonic pregnancies diminishes embryonic mortality between day 16 and the time when the embryo proper is clearly identifiable by ultrasound (days 31-42) to less than 10%. The manuscript also provides an update on novel tools used to elucidate embryonic metabolic and signalling requirements, as well as on the molecular regulation of key developmental landmarks that must be achieved during developmental windows most susceptible to pregnancy loss.

## Introduction

Pregnancy losses in cattle exert a major economic impact on farming profitability, mainly by increasing non-productive periods commonly known as open days in dairy cattle (De Vries, 2006). The magnitude of the economic loss associated to pregnancy failure depends on two major factors: the stage at which pregnancy loss occurs and the characteristics of the production system. Regarding to the first, pregnancy losses occurring before maternal recognition of pregnancy result in return to oestrus and thereby to a minimal increase in open days. In contrast, embryonic losses occurring after maternal recognition of pregnancy are associated to longer non-productive periods and may entail additional complications, such as increased risk of metritis, especially if they occur after implantation. The economic impact of pregnancy loss depends also on the specificities of the production system (e.g., dairy vs. beef, continuous vs. seasonal breeding).

**Figure 1:**
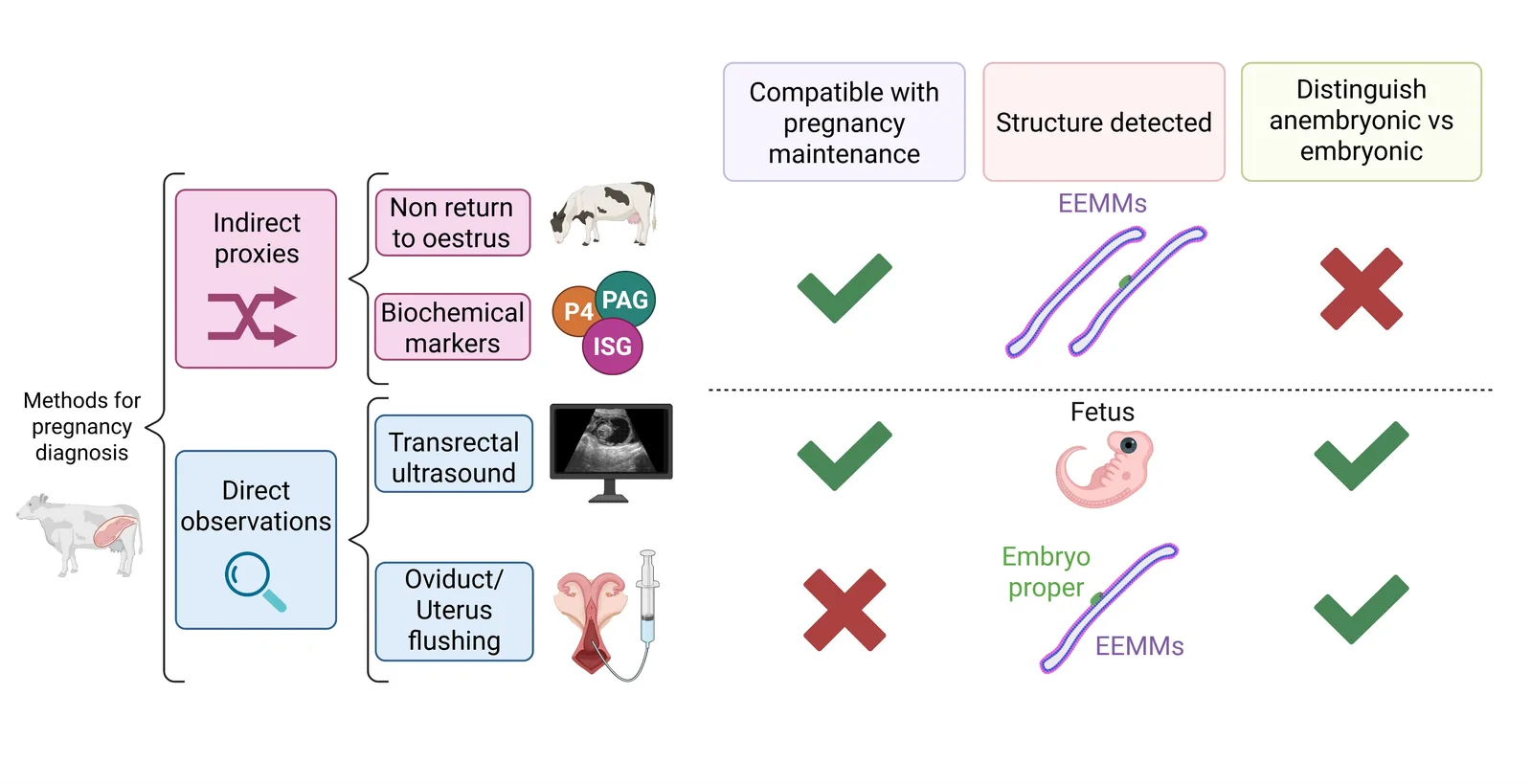
Overview of the methods to estimate or determine embryo survival. Indirect proxies inform about the survival of extra-embryonic membranes (EEMs), but cannot distinguish between embryonic and anembryonic pregnancies. In contrast, direct observations inform on the survival of the embryo proper but US cannot be performed before days 31-35 and embryo flushing is not compatible with pregnancy maintenance. Created in BioRender (2026).

To understand when pregnancy losses occur, two major approaches have been employed: direct observation of the embryo/fetus and indirect proxies of pregnancy, such as nonreturn to oestrus or the detection of biochemical markers (Figure 1). Direct observation of the embryo proper is achievable by transrectal ultrasound as early as day 20 of gestation, although given the small size of the embryo proper at this stage (~4 mm), the diagnose is more reliable around day 35, when it reaches ~2 cm in length (Curran et al., 1986). Before being detectable by ultrasound, direct observation requires oviductal or uterine flushing, an invasive intervention incompatible with pregnancy maintenance and thereby limited to experimental settings. In contrast, the diagnose by indirect proxies requires minimal intervention and do not interrupt pregnancy. However, they provide conflicting results to estimate pregnancy losses as they inform about the survival of the extra-embryonic membranes (EEMs), but not about the survival of the embryo proper. In other words, indirect proxies cannot distinguish between real pregnancies, where the embryo is still alive, and anembryonic pregnancies (pseudopregnancies), where EEMs develop in the absence of a viable embryo. This issue hinders the identification of the developmental origins of pregnancy loss around the period of pregnancy recognition and implantation.

Direct observation of the embryo or conceptus after embryo flushing also encompasses three limitations to understand the developmental origin of pregnancy loss. The first, already mentioned, is that embryo flushing is not compatible with pregnancy maintenance, which limits the number of studies that have directly assessed embryo viability at some stages, particularly after blastocyst hatching. Second, embryo flushing does not allow continuous observation of embryo development, providing only a snap shoot at a particular time point. As a consequence, pregnancy losses are estimated by flushing embryos in cohorts of animals at specific time points and, therefore, it is not possible to determine when developmental abnormalities first arise or whether embryos deemed viable at recovery will successfully progress. This information is critical to develop stage-specific reproductive, nutritional or pharmacological strategies to prevent embryo loss. Finally, and related to the previous limitation, when no structures or less structures than the number of corpora lutea observed in the ovaries are recovered, it is unclear whether embryos failed to develop or whether viable embryos were not retrieved after flushing. Acknowledging the limitations of direct and indirect estimations of pregnancy, this review integrates knowledge gathered from both descriptive approaches with functional *in vitro* and *in vivo* studies to shed light into the developmental failures causing pregnancy loss.

**Figure 2:**
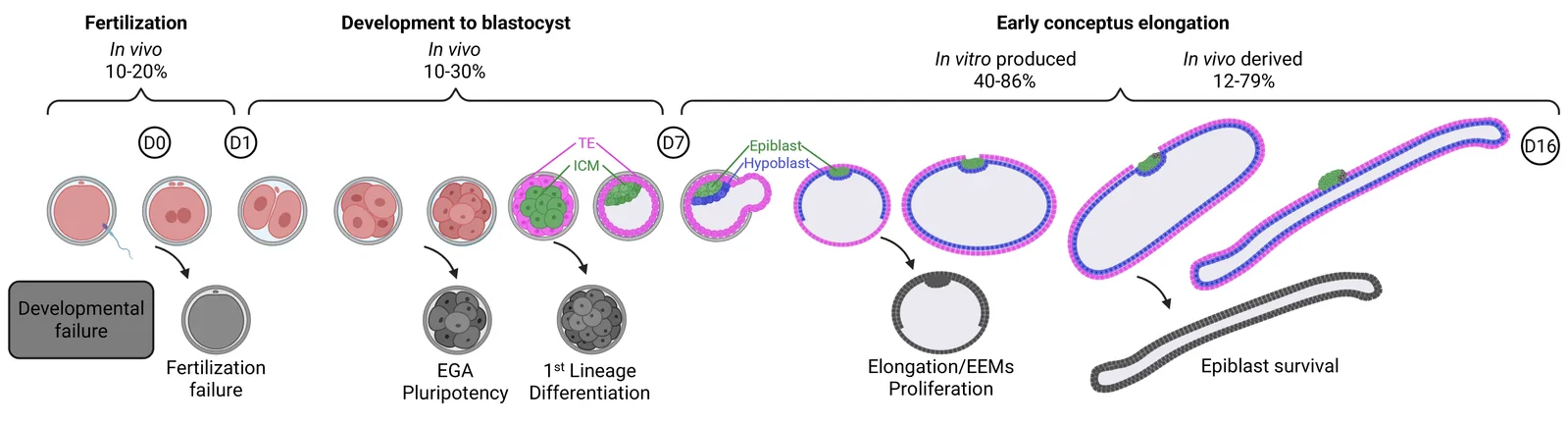
Pregnancy losses associated to developmental failures occurring from fertilization to maternal pregnancy recognition. The percentages depicted above indicate pregnancy losses calculated out of ovulated oocytes. In the case of fertilization failure and developmental arrest prior to blastocyst these percentages are based on the observation of structures recovered (Table 1). The percentage of pregnancy loss associated to embryonic mortality during early conceptus elongation integrates recovery rates and the incidence of embryo-devoid structures (Table 2). To provide data out of ovulated oocytes comparable to those provided for earlier failures, a ~30% pregnancy loss from ovulation to blastocyst has been assumed. Created in BioRender (2026).

Another gap in the knowledge of the root causes of pregnancy loss is the limited information about the molecular regulation and the nutritional and signalling requirements of embryo development in cattle. Technical constraints have historically limited gene ablation experiments to the mouse model (reviewed by Lamas-Toranzo et al., 2018b), leading to the assumption of an overtly conserved molecular regulation of embryo development across mammals which has been later proven to be wrong (reviewed by Pérez-Gómez et al., 2021a). Fortunately, the increasing use of gene editing techniques in cattle and other farm animals is starting to shed light into the molecular regulation of embryo development, particularly at prehatching stages. Similarly, the lack of *in vitro* systems to recapitulate embryo development following blastocyst hatching has hampered the continuous observation of embryo development and the elucidation of the nutritional and signalling requirements during this critical developmental period. Luckily, novel *in vitro* systems are currently available to achieve early posthatching embryo development in ruminants (reviewed by Martínez de los Reyes et al., 2026a). These novel tools will be also discussed and an updated view of the molecular regulation of embryo development will be provided at each specific section corresponding to different developmental periods. Figure 2 provides an overview on the topics that will be discussed in this review.

## Fertilization failure

Embryo development starts upon the fusion of two gametes during fertilization. Fertilization failure, estimated from the percentage of unfertilized structures obtained following flushing, averages 10 to 20% in cattle (Table 1, reviewed by Diskin and Morris, 2008; Sartori et al., 2009). While fertilization failure may be caused by impaired sperm functionality, either inherent to sperm or induced by a detrimental uterine and oviduct milieu, suboptimal oocyte quality seems to be the prevalent cause. A compelling example of the strong influence of oocyte quality on fertilization failure is illustrated by the well-documented negative effects of heat stress, which can increase fertilization failure rates to a catastrophic 45% despite the observation of sperm bound to the zona pellucidae of unfertilized oocytes, which evidences an apparently unaltered sperm functionality (Sartori et al., 2002). The negative effects of heat stress on fertilization rates are also observed in *in vitro* studies that exclude the potential effects of heat stress on sperm functionality by conducting fertilization and culture in controlled thermoneutral conditions (reviewed by Gómez-Guzmán et al., 2024). In line with this rationale, *in vitro* embryo production constitutes an effective reproductive management strategy to discard incompetent oocytes affected by heat stress and improve the pregnancy rates obtained by artificial insemination (Ambrose et al., 1999; Rutledge, 2001; Baruselli et al., 2020).

**Table 1:** Estimations of fertilization failure and embryonic arrest from zygote to blastocyst based on the observation of structures recovered. Data are presented as percentages of failures or losses per ovulated oocyte.

Reference	Fertilization failure	Zygote to blastocyst embryonic arrest	Bred
Spitzer et al. (1978)	18%	n/a	Beef
Diskin and Sreenan (1980)	10%	3-10%	Beef
Roche et al. (1981)	19%	0%	Beef
Smith et al. (1982)	7-13%	n/a	Beef
Breuel et al. (1993)	15-22%	n/a	Beef
Wiebold (1988)	0%	52%	Dairy
Sartori et al. (2002)	11-12%	21-44%	Dairy
Sartori et al. (2002)	0-45%	28-37%	Heat stressed dairy
Cerri et al. (2009a)	13-27%	23-36%	Dairy
Cerri et al. (2009c)	16-27%	20-34%	Dairy
Cerri et al. (2009b)	12-15%	14-45%	Dairy
Ribeiro et al. (2016)	17%	27%	Dairy
Berg et al. (2022)	16%	15%	Dairy
Crowe et al. (2025)	10%	16.5%	Dairy
Sartori et al. (2003)	21%	26%	Superovulated dairy
Wiltbank et al. (2016)	~10%	10-50%	Meta-analysis
Reese et al. (2020)	n/a	28%	Meta-analysis

The biological basis of fertilization failure in cattle is uncertain but can be associated to the ovulation of oocytes that have not fully reached developmental competence. Supporting this notion, superovulation -a procedure that entails the recruitment of oocytes that are not fully competent– has been associated to a significant increase in fertilization failure -up to 45%- compared with single ovulation (reviewed by Sartori et al., 2009). The molecular mechanisms involved in cattle fertilization have not been studied in detail, but they may be similar to those well studied in murine models. Indeed, the elucidation of the precise role of the protein TMEM95, the third sperm protein known to be essential for mammalian fertilization (Lamas-Toranzo et al., 2020; Noda et al., 2020), was based on the discovery of a mutation causing infertility in bulls (Pausch et al., 2014). On the oocyte side, JUNO is still the only protein known to be essential for mammalian fertilization (Bianchi et al., 2014), and its role seems to be conserved in cattle, where an *in vitro* assay based on JUNO protein coated beads was proposed as a test to predict bull fertility (Hamze et al., 2020).

## Developmental arrest at prehatching stages

Following fertilization, the embryo relies initially on the organelles, proteins and mRNA stored in the oocyte. The zygote undergoes a series of symmetrical cell divisions giving rise to initially totipotent blastomeres, but the gradual activation of the embryonic genome, spanning from the 4- to 16-cell stages in cattle (Graf et al., 2014), enables the appearance of transcriptional differences among individual blastomeres. Such blastomere-specific transcriptional regulation is required for the first lineage differentiation event, which relies on a cell-type specific transcriptional regulation triggered by mechanical clues: outer polar cells of the morula upregulate transcription factors leading to trophectoderm (TE) differentiation, whereas the transcriptional regulation of the inner apolar cells is reprogrammed to give rise to the inner cell mass (ICM) (Berg et al., 2011). TE differentiation confers specific functional properties to these cells, enabling the establishment of robust intercellular junctions and the influx of water, both of which are required to form the blastocoel, a defining characteristic of the blastocyst stage (reviewed by Kim and Bedzhov, 2022).

The incidence of embryonic arrest during prehatching embryo development of *in vivo* derived (IVD) embryos is estimated by the morphological evaluation of the structures flushed from the reproductive tract at specific time points. *In vivo* estimations vary greatly among studies, averaging 10 to 30% (Table 1, reviewed by Berg et al., 2022; Reese et al., 2020; Wiltbank et al., 2016). These estimations can be refined by *in vitro* observations, as this developmental period can be fully recapitulated by conventional *in vitro* embryo production systems. As in the case of fertilization failures, suboptimal oocyte competence constitutes a major cause for developmental arrest at prehatching stages. Functional evidence for the effect of oocyte quality on prehatching development was provided by pioneer studies that observed that, under the same *in vitro* fertilization (IVF) and culture (IVC) conditions, the rate of developmental arrest increases in oocytes matured *in vitro* compared to those matured *in vivo* (Dieleman et al., 2002; van de Leemput et al., 1999; Rizos et al., 2002). Employing optimized IVF and IVC conditions, about one third of the zygotes generated from *in vivo* matured oocytes fail to reach the blastocyst stage, whereas this proportion rises to roughly 50% for zygotes derived from *in vitro* matured oocytes, highlighting the need for improving current oocyte maturation systems (reviewed by Luciano et al., 2018).

**Figure 3:**
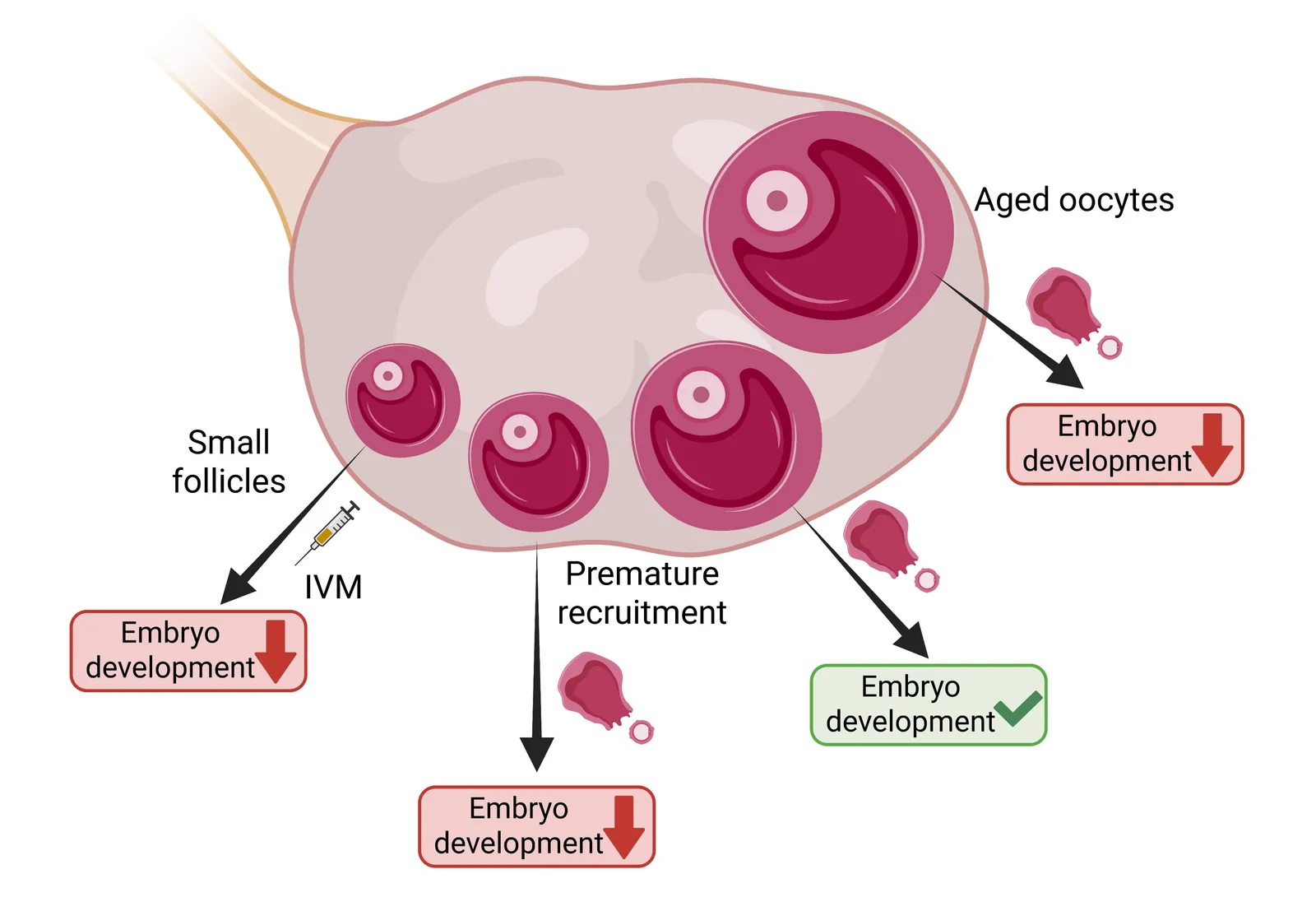
Impact of oocyte maturation status on embryo survival. Ovulation of too small or too large follicles and conducting the final phase of follicular development *in vitro* are associated with increased rates of embryonic arrest at prehatching stages. Created in BioRender (2026).

The specific molecular mechanisms leading to reduced oocyte competence remain unclear, despite the overwhelming number of descriptive studies reporting potential markers of oocyte competence in oocytes, cumulus cells or follicular fluid (reviewed by Oliveira et al., 2026). As nuclear maturation rates are often above 90% both *in vivo* and *in vitro* (Lonergan and Fair, 2016), ooplasm composition arises as the major driver of developmental competence to the blastocyst stage. Ooplasm components include proteins, mRNA and organelles that are required for embryo development to embryonic genome activation and beyond. As these critical ooplasm components are acquired and stored along follicular growth, suboptimal ooplasm composition may result from a failure of the tightly controlled coordination between ovulation and oocyte maturation (Figure 3, reviewed by Robker et al., 2018). In agreement, the ovulation of too small or too large follicles is associated to reduced pregnancy rates (Perry et al., 2007). On one hand, reduced developmental competence can arise from the ovulation of oocytes that have not completed cytoplasmic maturation, as evidenced by the lower developmental rates obtained following IVM from small follicles (Lonergan et al., 1994) or from those prematurely recruited by superovulation (reviewed by Sartori et al., 2009). On the other, aged oocytes from follicles overgrown under reduced circulating progesterone levels also show reduced developmental ability to reach the blastocyst stage (Ahmad et al., 1995; Cerri et al., 2009c; Revah and Butler, 1996). This rationale provides a biological basis for synchronization programs aimed to improve embryo development (Hayden et al., 2022; Seneda et al., 2020). Oocyte developmental competence can be also impaired by metabolic conditions (Carvalho et al., 2014; Leroy et al., 2005), opening the way for the development of nutritional strategies to improve fertility.

Among the ooplasm components critical for embryo development, mitochondria have drawn considerable attention in human assisted reproduction (Yildirim and Seli, 2024). As in human studies, the amount of mitochondria present in the bovine oocyte (Lamas-Toranzo et al., 2018a), but not that of its corresponding cumulus cells (Martínez-Moro et al., 2022), has been positively associated with its competence to reach the blastocyst stage. This correlation opens the way to therapies aimed at increasing mitochondrial amount or at improving their functionality to enhance oocyte competence. In this sense, whereas mitochondrial supplementation approaches developed in human assisted reproduction (Ferreira et al., 2021) are not practically applicable to cattle, nutritional strategies could be implemented (Cui et al., 2025).

Although oocyte competence is a major determinant of prehatching developmental success, the contribution of the oviductal and uterine environment should not be overlooked. Functional studies on this area are limited, as isolating the effect of the reproductive tract environment during prehatching development from oocyte competence inevitably requires oviductal embryo transfer, a technically challenging approach in cattle (reviewed by Besenfelder, 2025). Oviductal transfer of IVP embryos has proven that suboptimal prehatching environment impairs embryo development by comparing developmental rates following embryo transfer in post-partum cows vs. heifers (Rizos et al., 2010) and lactating vs. non lactating cows (Maillo et al., 2012), proving that a suboptimal prehatching environment reduces blastocyst rate.

## Molecular regulation of prehatching embryo development

Prehatching development is characterized by two critical developmental landmarks: embryonic genome activation (EGA) and first lineage differentiation. Critical genes involved in murine EGA include DUX4 (Hendrickson et al., 2017; De Iaco et al., 2017; Whiddon et al., 2017), NFYA (Bhattacharya et al., 2003; Lu et al., 2016), YAP1 (Yu et al., 2016), and OBOX (Ji et al., 2023), but the molecular regulation of murine EGA may not be conserved in cattle (reviewed by Halstead et al., 2020). The essential role of DUX4 in EGA appears to be conserved, as its downregulation significantly reduces bovine blastocyst formation (Halstead et al., 2022). Additional functional studies have identified other genes essential for prehatching development in cattle. For instance, COPA knock-out (KO) bovine embryos arrest their development before the 8-cell stage (Miskel et al., 2025), ZSCAN4 downregulation was reported to induce embryonic arrest before the 16-cell stage (Takahashi et al., 2019), and pharmacological inhibition or ablation of the transcription factor SP1 causes morula arrest in bovine embryos (Jin et al., 2026; Talukder et al., 2025). In any case, it must be highlighted that failures in EGA are not the only root cause of developmental arrest prior to the blastocyst stage (Figure 2). Early cleavage arrest, occurring before or around EGA, can be also linked to loss of totipotent-like features, as evidenced by the ablation of OCT4 in cattle embryos (Daigneault et al., 2018) and own unpublished results). Developmental arrest beyond EGA (i.e., late cleavage/morula stages) is more likely to be due to failures in first lineage differentiation, caused by either direct impairment of the molecular mechanisms involved in the process or by general failures in embryonic homeostasis or metabolism.

Blastocyst formation relies on first lineage differentiation, which confers the outer TE cells of the morula the ability to form the blastocoel. The molecular regulation of first lineage differentiation is well studied in mice, where Hippo pathway becomes inactive in the TE precursors leading to a TE-specific transcriptional program driven by the transcription factor TEAD4 (Nishioka et al., 2008). However, in contrast to mice, functional studies have uncovered that TEAD4 is dispensable for blastocyst formation and TE development to elongated stages in cattle (Pérez-Gómez et al., 2021b, 2024b; Wu et al., 2024). Similarly, downstream effectors of TEAD4 in mice are not required for TE differentiation in cattle. EOMES, essential for murine TE differentiation (Russ et al., 2000) is not expressed at the bovine blastocyst stage (Berg et al., 2011), and CDX2, another critical TE regulator in mice (Strumpf et al., 2005) is not required for blastocyst formation in cattle, as shown by both downregulation (Goissis and Cibelli, 2014) and ablation (Shi et al., 2023) studies.

Given the strikingly low conservation of the molecular mechanisms responsible for first lineage differentiation between mice and cattle, recent efforts have focused on identifying the transcription factors involved in this process in cattle and other ungulates. A recent study reported that TEAD4 and TEAD3 may play redundant functions in TE differentiation, as the ablation of TEAD4 combined with the downregulation of TEAD3 impaired blastocyst formation (Yu et al., 2024). Beyond TEAD factors and CDX2, TE-specific transcription factors have been investigated. Gene ablation studies have found that GATA3 is dispensable for blastocyst formation in cattle and sheep (Martínez de los Reyes et al., 2025; Shi et al., 2023), whereas downregulation or ablation of TFAP2C disrupts blastocyst formation in pigs and sheep (Martínez de los Reyes et al., 2026b; Zhang et al., 2024).

## Developmental failures from blastocyst hatching to maternal recognition of pregnancy

Following blastocyst formation, expansion of the blastocoel eventually leads to zona pellucida rupture and blastocyst hatching. In rodents and humans, implantation occurs shortly after blastocyst hatching, but in ungulates blastocyst hatching inaugurates a second period of pre-implantation embryo development termed conceptus elongation. Conceptus elongation can be subdivided into two phases, the first spanning from blastocyst hatching to maternal recognition of pregnancy, and the second spanning from maternal recognition of pregnancy to implantation. In cattle, maternal recognition of pregnancy occurs by day 16, as although luteolysis starts by day 18 or 19, pioneer *in vivo* functional studies observed that embryo transfer in cyclic cows by day 16, but not at day 17, prevents luteolysis (Betteridge et al., 1980) and -conversely- embryo removal after day 16, but not earlier, results in an extended luteal phase (Northey and French, 1980).

Before maternal recognition of pregnancy, the embryo must accomplish a series of cell differentiation, proliferation and migration processes (reviewed by Artus et al., 2020; Pérez-Gómez et al., 2021a). Around blastocyst hatching, the ICM differentiates into the epiblast, which will form the embryo proper, and the hypoblast, a second extra-embryonic lineage also known as primitive endoderm (Figure 2). The hypoblast will subsequently proliferate and migrate lining the inner side of the TE (i.e., that facing the blastocoel) and forming the bilaminar structure (TE + hypoblast) that characterizes the extra-embryonic membranes (EEMs). EEMs proliferate extensively shaping the conceptus from spherical to ovoid, tubular and finally filamentous. Meanwhile, the epiblast forms an embryonic disc (ED) which develops to the early streak phase of gastrulation by day 15, while EEMs are starting to signal pregnancy (van Leeuwen et al., 2015).

**Figure 4:**
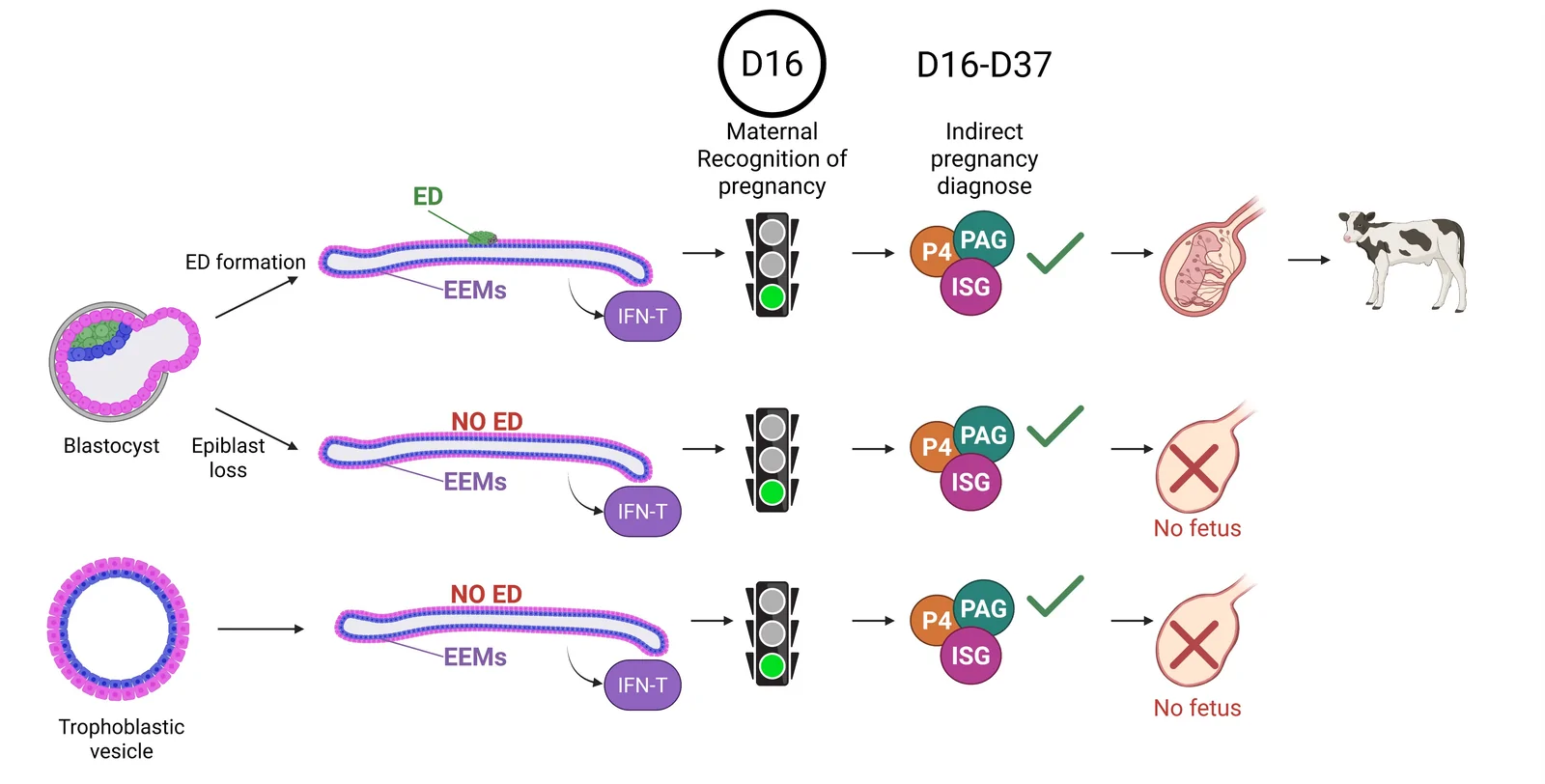
Maternal recognition of pregnancy does not require survival of the embryo proper. Embryo-devoid structures formed by extra-embryonic membranes, developed naturally or by the generation of trophoblastic vesicles, can prevent luteolysis up to day 37, resulting in anembryonic pregnancies. Created in BioRender (2026).

The estimation of the embryonic losses occurring during this period and the beginning of the following one are particularly challenging due to the inaccessibility of the developing conceptus. As the small size of the embryo proper (i.e., the ED) prevents its reliable identification by ultrasound, direct observation requires uterine flushing and the identification of the ED in the recovered conceptus. Besides, as embryos arrested at prehatching or early posthatching stages are often degraded by the uterine environment before flushing, survival rate is calculated based on recovery rate, assuming that no viable conceptus remain in the uterus after flushing, a premise difficult to verify when flushing is conducted *in vivo*. In contrast, indirect proxies of pregnancy, such as nonreturn to oestrus or biochemical markers only inform about the development of the EEMs, which, as it will be discussed below, are not indicative of the survival of the embryo proper (Figure 4).

Indirect proxies of pregnancy rely on signalling involved in maternal recognition of pregnancy, which is largely mediated by the secretion of interferon tau (IFNT) in ruminants (Helmer et al., 1989; Imakawa et al., 1987), although a recent study -currently under revision- has reported that cattle embryos do not require IFNT to signal pregnancy (Wolf et al., 2026 forthcoming). Importantly, IFNT can be expressed by the EEMs in the absence of an ED, as evidenced by a pioneer study that observed that the transfer of “trophoblastic vesicles”, i.e. 0.2 to 4 mm spheres formed upon culture of fragments of EEMs obtained from elongated conceptuses, were sufficient to maintain corpora lutea and extend oestrus cycle up to day 37 (Heyman et al., 1984). A later study reported similar extension of the luteal phase (33-35 days inter-oestrous interval) after transferring blastocyst-derived trophoblastic vesicles (probably formed only by TE) (Nagai et al., 2009). These functional studies evidence that the control of the maintenance of the corpora lutea is not embryonic but extra-embryonic up to around 2 weeks after maternal recognition of pregnancy (Figure 4). As a result, indirect proxies such as nonreturn to oestrous, the detection of interferon stimulated genes in peripheral blood cells by day 18 (Green et al., 2010; Stevenson et al., 2007) or TE-produced pregnancy associated glycoproteins by day 25 (Green et al., 2000) are unable to distinguish anembryonic pregnancies (pseudopregnancies) from viable pregnancies.

The incidence of the pregnancy losses from blastocyst to maternal recognition of pregnancy have been estimated mainly by indirect approaches (reviewed by Reese et al., 2020; Wiltbank et al., 2016). However, considering the limitations of indirect approaches, these estimations could underestimate the embryonic losses at this period and overestimate the embryonic losses at the following one. The percentage of IVD structures recovered at elongated stages lacking ED varies greatly from 0 to 63%, which in some cases rises considerably the mortality rate (12-79%) previously calculated based on indirect proxies or on the presence of elongated structures irrespective of the presence of the embryo proper (Table 2). Developmental arrest due to lethal haplotypes constitutes special cases of pregnancy failure in IVD embryos. Dedicated investigation of the developmental arrest caused by two of them (HH3 and HH5) has revealed that double carrier embryos arrest their development during posthatching development and prior to maternal recognition of pregnancy (Pérez-Gómez et al., 2024d, 2024a).

**Table 2:** Estimations of embryonic mortality from blastocyst to early elongated stages, before maternal recognition of pregnancy (day 16). Total embryonic loss is calculated integrating the rate of structures not recovered (with or without ED) and the percentage of structures lacking ED.

Reference	Type of embryo	Structures lost from blastocyst to early elongation	Structures lacking ED (%)	Total embryonic loss
Diskin and Sreenan (1980)	AI	n/a (38% not recovered from AI)	n/a	n/a
Smith et al. (1982)	AI	n/a (22% not recovered from AI)	n/a	n/a
Carter et al. (2008)	AI	n/a (50% not recovered from AI)	n/a	n/a
Berg et al. (2022)	AI	10%	5%	15%
Pérez-Gómez et al. (2024b)	AI	n/a	0%	n/a
Pérez-Gómez et al. (2024a)	AI	n/a	0%	n/a
O’Hara et al. (2014a)	AI	n/a (42% not recovered from AI)	n/a	n/a
	IVP-ET	59%	n/a	n/a
Rexroad and Powell (1999)	IVP-ET	20-65%	20-50%	40-63%
	IVD-ET	n/a	21%	n/a
Bertolini et al. (2002)	IVP-ET	39%	65%	79%
	IVD-ET	44%	63%	79%
Ideta et al. (2007)	IVP-ET	40%	21%	53%
	IVD-ET	4%	8%	12%
Alexopoulos et al. (2008)	IVP-ET	75%	45%	86%
	Cloning-ET	85-93%	55-63%	93-97%
Rodríguez-Alvarez et al. (2010)	IVP-ET	45%	17%	54%
	Cloning-ET	27%	37%	54%
Smith et al. (2010)	IVP-ET	22%	34%	49%
	Cloning-ET	31%	26%	49%
Fischer-Brown et al. (2004)	IVP-ET	58-69%	61-27%	77-84%
Block et al. (2007)	IVP-ET	70%	24%	77%
Berg et al. (2010)	IVP-ET	49%	17%	58%
Loureiro et al. (2011)	IVP-ET	34	17%	45%
O’Hara et al. (2014b)	IVP-ET	43%	n/a	n/a
Desmet et al. (2020)	IVP-ET	68%	27%	77%

*In vitro* produced (IVP) embryos experience a 10 to 40% higher rate of pregnancy loss compared to IVD, mainly due to developmental failures during this period (reviewed by Ealy et al., 2019; Sartori et al., 2025). The percentage of IVP elongated structures lacking embryonic disc oscillates between 17 and 65%, being generally higher than those attributed to IVD embryo (Table 2). Combining the percentage of ED-devoid structures with recovery rate (which accounts both conceptus and ED-devoid structures), embryo mortality rate during this period oscillates between 40 and 86% (Table 2). These embryo-devoid structures frequently observed in IVP embryo transfers can cause an apparent increase in pregnancy loss at later stages (e.g., days 28 to 40 in Sartori et al., 2025), as pregnancy diagnose at day 28 do not discriminate between embryonic and anembryonic pregnancies.

## Lineages development during early conceptus elongation

During early conceptus development, the EEMs, which are responsible for pregnancy signalling, form following hypoblast migration underneath the TE and undergo massive proliferation (Figure 2). Simultaneously, the epiblast must form an ED and start gastrulation. Among these processes, the most vulnerable are those pertaining the epiblast, as evidenced by the frequent observation of structures only formed by functional extra-embryonic lineages (Table 2). *In vitro* observations further highlight the vulnerability of the epiblast, as initial posthatching bovine embryo culture systems failed to maintain such lineage (Brandão et al., 2004). Although more advanced systems support the development of EEMs, epiblast survival rate remains limited to approximately 50% (Ramos-Ibeas et al., 2020), and *in vitro* development beyond early ED stages (i.e., the beginning of gastrulation) has been only achieved in sheep embryos upon the addition of specific cytokines (Ramos-Ibeas et al., 2023). Given the critical role of epiblast degeneration in pregnancy loss following embryo transfer of *in vitro* produced embryos (Table 2, reviewed by Ealy et al., 2019), different prehatching media compositions have been tested to enhance the prospective survival of the epiblast (Oliver et al., 2025; Ramos-Ibeas et al., 2023; Speckhart et al., 2024).

**Figure 5:**
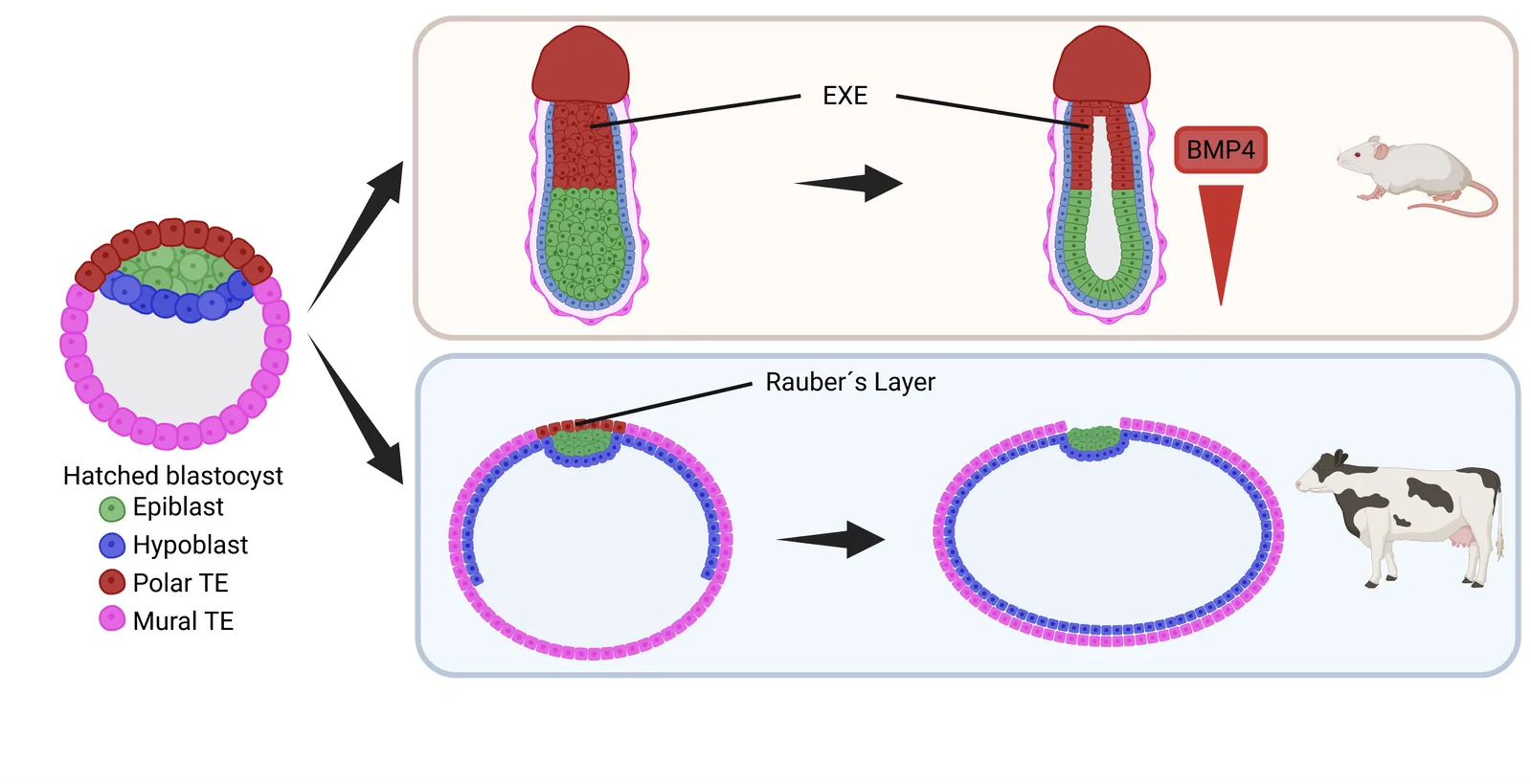
Differences in posthatching embryo development between mice and cattle. Murine polar TE forms the extra-embryonic ectoderm (EXE), which expresses BMP4, a secreted protein required for the development of the epiblast-derived tridimensional egg cylinder and symmetry breaking. In contrast, cattle polar TE forms the Rauber layer, which disappears well before symmetry breaking starts in the epiblast-derived flat embryonic disc. Created in BioRender (2026).

The molecular basis of epiblast development around peri-gastrulation has been well characterized in mice, which unfortunately exhibits remarkable developmental differences compared with other mammals (Figure 5). Murine epiblast develops into a cup-shaped egg cylinder and is dependent on different signalling molecules produced by both TE and hypoblast derivatives. In brief, polar TE (i.e., that covering the ICM) proliferates extensively forming the extra-embryonic ectoderm (ExE). ExE expresses BMP4, a secreted protein required for epiblast development (Winnier et al., 1995). The tridimensional geometry of the adjacent epiblast-derived egg cylinder allows the generation of a BMP4 gradient that initiates symmetry breaking by triggering the appearance of the hypoblast-derived distal visceral endoderm (Yamamoto et al., 2004). In contrast to mice, the epiblast of most mammals (including ungulates, humans or rabbits) forms a flat ED where the well characterized tridimensional signalling gradients governing mouse gastrulation are not geometrically possible (Pfeffer, 2022). Furthermore, ungulate embryos do not develop ExE, as their polar TE -termed Rauber´s layer- disappears by Day 12 in cattle (van Leeuwen et al., 2020), well before the initiation of gastrulation occurring at Day 14. Interestingly, beyond the morphological similarities between human and cattle EDs, the human epiblast is also particularly vulnerable to developmental failures. Impaired epiblast development during human peri-gastrulation results in implanted anembryonic structures termed “blighted ova”, which constitute the single leading cause of human miscarriage (Chaudhry et al., 2026) and share the same developmental origin than anembryonic pregnancies in cattle.

Given the unknown molecular regulation of epiblast development in cattle, it remains unclear whether inter-lineage signalling is required for ED development or -conversely- if ED development promotes the development of EEMs. In this sense, although the growth of trophoblastic vesicles proves that the ED is not required for EEMs development, it is unclear if EEMs proliferate to the same extent in a conceptus than in a structure lacking an ED. This is relevant, as given that the conceptus must reach a minimal length (~5 mm) to produce enough IFNT to signal pregnancy (Mann and Lamming, 2001), the concept “bigger is better” has been routinely employed to test conceptus viability, ignoring in many cases the presence or absence of the ED. To answer that question, we generated epiblast devoid structures by gene editing observing that epiblast-containing and epiblast-devoid structures elongate to a similar extent in the uterus (unpublished observations, preliminary results can be found at Flores-Borobia et al., 2026).

## The influence of the uterine environment on posthatching embryonic loss

The clear effect of prehatching embryo culture conditions on subsequent embryo survival evidences that posthatching embryonic loss can arise from carry over effects on the development of the epiblast, discussed in the next section. However, the diverging developmental outcomes obtained after transferring morphologically similar blastocysts to different recipients suggests that the uterine environment is also a major determinant for embryo survival. The uterine fluid is a complex mix of nutrients, hormones and growth factors supplied from the selective transport of blood components and the secretions of the uterine glands, which were proven to be essential for conceptus elongation in ewes (Gray et al., 2001). The composition of proteins and metabolites of the uterine fluid is dynamic and adapts to the changing demands of the conceptus (e.g., (Forde et al., 2014; González-Brusi et al., 2026; Hugentobler et al., 2007; Simintiras et al., 2019), and therefore suboptimal uterine fluid composition can cause embryonic arrest. Knowing the metabolic and signalling requirements of the conceptus would facilitate the rational development of nutritional or pharmacological strategies to prevent embryonic loss but, as most of the information available is descriptive, these requirements remain elusive.

The advances on *in vitro* systems and genome editing have started to shed light into the conceptus requirements. The development of culture media where embryos can proceed through the initial phases of conceptus elongation (reviewed by Martínez de los Reyes et al., 2026a) allows testing the effects of the addition of specific compounds. This strategy has served to uncover the dispensable role of arachidonic acid during early elongation (González-Brusi et al., 2023) and to discover the developmental roles of specific signalling pathways such as TGFβ (Galiano-Cogolludo et al., 2023) or MEK (Martínez de los Reyes et al., 2024). The incipient development of uterine organoids (Devkota et al., 2026; Edge et al., 2026) may also serve in the future to test strategies to modify the uterine gland secretions on controllable *in vitro* settings. Finally, the development of efficient methods to knock-out genes in cattle embryos (Lamas-Toranzo et al., 2019) possibilities functional studies where a metabolic or signalling route is ablated genetically. This strategy has been used to determine that embryonic PPARG is dispensable for cattle embryo development to day 14 (Pérez-Gómez et al., 2024c), suggesting that the impaired conceptus elongation observed in ewes where PPARG was depleted in both embryo and uterus, is mediated by the uterine PPARG (Brooks et al., 2015).

## Embryonic and fetal mortality beyond maternal recognition of pregnancy

Following maternal recognition of pregnancy, bovine EEMs initiate apposition for implantation at day 17 (King et al., 1981) and continue growing to occupy both uterine horns and initiate implantation by day 21 (Bazer et al., 2009; Guillomot, 1995). Concurrently, the ED progresses through gastrulation, which differentiates the epiblast into endoderm, mesoderm and ectoderm lineages, and undergoes paraxial, intermediate and lateral plate mesoderm differentiation and by day 21, with the most advanced embryos displaying neural tube, somites and allantoid development (Maddox-Hyttel et al., 2003).

The period consecutive to maternal recognition of pregnancy probably constitutes the most difficult one to assess embryo development and survival, as embryos remain undetectable by ultrasound and their increased length initially and implantation status later impede *in vivo* collection by uterine flushing. For that reason, pregnancy estimations before days 31-35 have been mostly based on indirect proxies of pregnancy such as nonreturn to oestrus and biochemical markers. As explained above, these estimations probably include a significant part of the embryonic losses occurring at earlier stages leading to anembryonic pregnancies, given that embryo-devoid EEMs are able to survive and prolong the interoestrous interval up to day 37 (Heyman et al., 1984). In agreement, while pregnancy loss estimations between days 28 and 60 average 12% (Wiltbank et al., 2016), pregnancy losses after reliable detection of the embryo proper (i.e., from day 32 to 60) were estimated to be around 6% (Reese et al., 2020). In coincidence, continuous observation of the embryo proper by ultrasound has served to estimate that about ~10% of the viable embryos at day 31 following AI do not survive to day 60 (Pohler et al., 2016), a figure that translated to embryonic losses out of the total oocytes roughly equals the 6% estimated by meta-analysis (Reese et al., 2020). The same study observed that IVP embryos experience a slightly higher embryonic loss from day 31 to day 60 after IVP (~11% out of total oocytes) and a large 20% drop in pregnancy rate between day 28 (where the diagnose does not discriminate between embryonic and anembryonic pregnancies) and day 31 (detection of the embryo proper) (Pohler et al., 2016). In summary, the deviations between pregnancy estimations from indirect proxies and those based on the observation of the embryo proper roughly coincide with the percentage of conceptuses lacking embryonic disc that result in false positive pregnancy diagnoses (Table 2).

After implantation, there is a gradual development of cotyledons and embryonic structures that finally evolves into a foetus at around day 42 (reviewed by Assis et al., 2009). Although entailing significant economic cost, foetal losses are uncommon (5-7%) and usually attributed to infectious diseases and genetic abnormalities (reviewed by Albaaj et al., 2023; Silke et al., 2002; Wiltbank et al., 2016).

## Concluding remarks

Pregnancy losses in cattle can be caused by failures in embryo or foetus development at any stage, but most of them occur due to embryonic mortality before maternal recognition of pregnancy (Figure 2). From ovulation to blastocyst formation, fertilization failures and embryonic developmental arrest combined constitute the major cause of overall pregnancy failure in IVD embryos. Nevertheless, a significant proportion of embryos reaching the blastocyst stage fail to develop into an elongated conceptus able to signal pregnancy. Other blastocysts are able to develop into structures formed by functional extra-embryonic membranes but fail to form an embryonic disc before maternal recognition of pregnancy. These embryo-devoid structures can derive from both IVD and IVP blastocysts, although the incidence of epiblast failure is higher in IVP blastocysts, thereby constituting a prevalent cause of pregnancy failure following embryo transfer. Given that embryo-devoid elongated structures can prevent luteolysis up to day 37 (Figure 4), they produce anembryonic pregnancies which, upon resumption, are mistakenly identified as late embryonic losses while their developmental origin roots to impaired epiblast development before maternal recognition of pregnancy.

## Data Availability

No research data was used.
